# The Multimodal Go-Nogo Simon Effect: Signifying the Relevance of Stimulus Features in the Go-Nogo Simon Paradigm Impacts Event Representations and Task Performance

**DOI:** 10.3389/fpsyg.2018.02011

**Published:** 2018-10-25

**Authors:** Thomas Dolk, Roman Liepelt

**Affiliations:** ^1^Department of Psychology, University of Regensburg, Regensburg, Germany; ^2^Institute of Psychology, German Sport University Cologne, Cologne, Germany

**Keywords:** stimulus-response compatibility, go-nogo Simon task, modality, event representations, referential coding, Theory of Event Coding

## Abstract

Numerous studies have shown that stimulus-response-compatibility (SRC) effects in the go-nogo version of the Simon task can be elicited as a result of performing the task together with another human or non-human agent (e.g., a Japanese-waving-cat, a working-clock, or a ticking-metronome). A parsimonious explanation for both social and non-social SRC effects is that highlighting the spatial significance of alternative (non-/social) action events makes action selection more difficult. This holds even when action events are task-irrelevant. Recent findings, however, suggest that this explanation holds only for cases of a modality correspondence between the Simon task as such (i.e., auditory or visual) and the alternative (non-/social) action event that needs to be discriminated. However, based on the fact that perception and action are represented by the same kind of codes, an event that makes the go-nogo decision more challenging should impact go-nogo Simon task performance. To tackle this issue, the present study tested if alternative stimulus events that come from a different sensory modality do impact SRC effects in the go-nogo version of the Simon task. This was tested in the presence and absence of alternative action events of a human co-actor. In a multimodal (auditory–visual) go-nogo Simon paradigm, participants responded to their assigned stimulus – e.g., a single auditory stimulus while ignoring the alternative visual stimulus or vice versa – in the presence or absence of a human co-actor (i.e., joint and single go-nogo condition). Results showed reliable SRCs in both, single and joint go-nogo Simon task conditions independent of the modality participants had to respond to. Although a correspondence between stimulus material and attention-grabbing event might be an efficient condition for SRCs to emerge, the driving force underlying the emergence of SRCs rather appears to be whether the attentional focus prevents or facilitates alternative events to be integrated. Thus, under task conditions in which the attentional focus is sufficiently broad to enable the integration and thus cognitive representation of alternative events, go-nogo decisions become more difficult, resulting in reliable SRCs in single and joint go-nogo Simon tasks.

## Introduction

In the last 15 years, cognitive scientists have invested much effort into investigating how and to what extent people mentally represent their own and other people’s actions/tasks and how these cognitive representations influence an individual’s own behavior when interacting with another person. The most prominent paradigm of this line of research is widely known as the joint Simon paradigm, in which two people share the standard version of the Simon task (Sebanz et al., 2003).

In the standard Simon task, single participants execute spatially defined actions in response to non-spatial stimulus features (e.g., “Press right in response to the high-pitched tone and press left in response to the low-pitched tone). Critically, however, both tones randomly appear to the left and the right of participants, leading to trials of spatially compatible and spatially incompatible stimulus-response (S-R) assignments (i.e., a high-pitched tone presented to the right side of the participant would be compatible, whereas the same tone presented to the left would be incompatible). Note that although stimulus locations are entirely task-irrelevant, they automatically activate spatially corresponding responses (i.e., the spatial location of the stimulus primes the response on the same side of space). In the case of a spatial match between the automatically activated and the assigned response, task performance is facilitated, whereas performance is impaired in the case of a spatial mismatch (Kornblum et al., 1990). This stimulus-response compatibility (SRC) effect, also known as the Simon effect (Simon, 1969; for reviews, see Proctor and Vu, 2006; Rubichi et al., 2006; Hommel, 2011), does typically not occur if the task is turned into a go-nogo task by having the participant execute single key presses in response to only a specific stimulus feature (i.e., a single tone/color; Hommel, 1996). However, an SRC re-emerges if the participant shares the same go-nogo task with another participant who responds to the other stimulus by operating the other response key–a phenomenon known as the social/joint SRC (Sebanz et al., 2003).

Such joint action effects have been taken to suggest that interacting individuals do not only form a cognitive representation of their own action or task but also (co-) represent the action or task of their co-actor (Sebanz et al., 2003, 2005; Welsh, 2009; Welsh et al., 2013; van der Wel and Fu, 2015). Co-representation is considered to be automatic and mandatory social in nature (Knoblich and Sebanz, 2006; Schmitz et al., 2017), such that the joint Simon task re-introduces a functionally similar kind of response competition as the standard Simon task (Kornblum et al., 1990; Sebanz et al., 2003). Recently, however, an increasing number of studies have challenged a purely social interpretation of SRC effects (e.g., Guagnano et al., 2010; Dolk et al., 2011, 2013; Dittrich et al., 2012, 2013; Sellaro et al., 2015; Stenzel and Liepelt, 2016; Michel et al., 2018). Some studies also provided evidence against a functional equivalence between the joint Simon task and the standard Simon task (Liepelt et al., 2011; Klempova and Liepelt, 2016). In line with these findings, Dolk et al. (2013) showed that the presence of another responding person is not required for (joint) SRC-like effects to occur. The presence of non-human “co-actors,” such as a Japanese waving cat, a clock, and a metronome, elicited SRCs that were comparable in size to the SRCs typically found when two people perform a go-nogo Simon task together (e.g., Sebanz et al., 2003; Guagnano et al., 2010; Liepelt et al., 2011; Welsh et al., 2013). Thus, response competition in a go-nogo Simon task may not be driven by the presence of another person performing a task-related action, but rather by the presence of another attention-grabbing action event during the task processing. According to the Theory of Event Coding (TEC; Hommel et al., 2001, Hommel et al., 2009) actions are cognitively represented by codes of their sensory consequences that are shared between self- and other-generated actions. Therefore, action control faces a discrimination problem between self-related event representations and simultaneously externally activated (non-self-related) event representations (Dolk et al., 2013). However, the exact nature of this action discrimination problem is not yet understood.

Studies analyzing the sequential modulation of Joint and Solo go-nogo SRC effects (Liepelt et al., 2011, 2013; Yamaguchi et al., 2018b) suggest that the relevant decision in the joint Simon task is a decision between the own go stimulus and the nogo stimulus (=go stimulus of the partner). When the go-nogo decision has to be performed together with a joint action partner, the presence of additional events due to the response of the partner during the nogo processing may enhance the relevance of the nogo stimulus, via a process that has been termed nogo tagging (Liepelt et al., 2011). In line with this idea, Baess and Prinz (2015) showed a modulation of stimulus processing as indicated by the Go- and NoGo-N1 component of the electroencephalogram (EEG). The modulation of the nogo decision by the presence of the responding partner has been interpreted as a change in agent identification – my turn vs. your turn (Liepelt et al., 2011; Wenke et al., 2011; Baess and Prinz, 2015). Based on the assumption that the presence of additional events during nogo processing enhances the task relevance of these events (Liepelt et al., 2011), we hypothesize that the presence of additional events during nogo processing may make it more difficult to discriminate between go and nogo processing (Kühn and Brass, 2010a,b; Weller et al., 2017). However, up to now, studies targeting SRCs in go-nogo versions of the Simon task either concentrated on manipulating the nature of alternative (social or non-social) action events (Tsai and Brass, 2007; Tsai et al., 2008; Lam and Chua, 2010; Müller et al., 2011; Stenzel et al., 2012, 2014; Dolk et al., 2013; Stenzel and Liepelt, 2016; Klempova and Liepelt, 2017) or varied the presence or absence of the response event by means of a responding partner (Sebanz et al., 2003; Welsh et al., 2007; Atmaca et al., 2011; Sellaro et al., 2013). To our knowledge no previous study has tested the impact of additional stimulus events on joint task performance. This is, however, a theoretically important question, as referential coding (Dolk et al., 2013) and TEC (Hommel et al., 2001) accounts would assume that perception and action are cognitively represented by the same kinds of codes (Prinz, 1997) and therefore alternative stimulus events that are present during the go-nogo decision should increase the difficulty of the discrimination problem. If this is true, this would indicate that joint go-nogo effects are driven not by the social context and co-representation of the action or task producing an agent discrimination conflict, but rather by concurrently activated stimulus or response events increasing the difficulty of the actor’s own go-nogo decision (Kühn and Brass, 2010a,b).

Two recent studies testing the joint go-nogo effect using event-producing non-social objects found reliable SRC effects for the auditory modality using an auditory go-nogo Simon task when a Japanese waving cat provided visual waving cues and auditory cues (Puffe et al., 2017; Lien et al., 2016). These studies, however, did not find reliable SRC effects in a visual go-nogo Simon task when using the same objects. Due to this asymmetry, Puffe et al. (2017) suggested that the correspondence between the attention-attracting event and the stimulus material of the Simon task determines whether or not an SRC is present. However, a visual task may have focused visual attention to the visual stimuli on the screen, which would also explain why subjects did not perceive the event-producing object placed on the table before the screen. The auditory stimuli where presented via two laterally located loudspeakers with a distance of about one meter, which could have broadened the attentional, focus bringing back the event-producing object into the attentional focus.

In the present study, we therefore tested the impact of stimulus and response events concurrently present during the go-nogo decision on single (single condition) and joint Simon task (joint condition) performance. Due to the previously observed asymmetry of task modality and the externally activated (task-irrelevant action/stimulus) event (Puffe et al., 2017), we also manipulated the modality of the go-nogo Simon task. By presenting the additional (task-irrelevant) event at the same location as the task relevant stimulus, the width of the attentional focus was held constant. This was done to test if the presence of the SRC in the go-nogo Simon task is due to (a) a modality correspondence between the attention-attracting event and the stimulus material or (b) a broadening of the attentional focus to integrate alternative (action and/or stimulus) events.

We predicted that if the integration of alternative events within the attentional focus and the corresponding enhanced difficulty of response discrimination underlie the SRC in the go-nogo Simon task, we should find a SRC effect in the presence of alternative events in Single visual and auditory go-nogo task conditions. Effects for both modalities should be larger when a concurrent response event is additionally present in the joint condition. In contrast, if the SRC effect is due to the modality correspondence of the attention-attracting event and the stimulus material, we should not find an SRC effect in Single visual and auditory go-nogo task conditions. That is because alternative events in our study are always presented in a different modality. Naturally, effects should be present in the joint condition in both visual and auditory modality conditions, as the co-actors response contains both visual and auditory information.

## Materials and Methods

### Participants

G^∗^Power 3.1 software (Faul et al., 2009) revealed that a sample size of N = 32 is required to guarantee sufficient statistical power of 1-β = 0.80 with α = 0.05, and partial η^2^ = 0.23 (Iani et al., 2011, Experiment 2). Based on this analyses and aiming to extend the classical finding of Sebanz et al. (2003) with 40 participants to a multimodal go-nogo Simon paradigm, we tested *N* = 40 participants (28 female; *M*_age_ = 23.5, *SD*_age_ = 2.8, *R*_age_ = 18–29 years). This guaranteed sufficient statistical power and compensates for potential dropouts in participants. Participants had no history of neurological or hearing problems. They were all right-handed as assessed by the Edinburgh Inventory (Oldfield, 1971; *M*_LQ_ = 92.8, *SD*_LQ_ = 8.3, *R*_LQ_ = 80–100), were naive with regard to the hypothesis of the experiment and were paid for their participation. Participants gave their written informed consent before their inclusion in the study in accordance with the ethical standards of the German Psychological Society (DGPs; 2016) and the 1964 Declaration of Helsinki. According to the DGP’s ethics commission, an institutional research board’s ethical approval is required only if (i) research carries additional risk beyond daily activities or (ii) any funding is subject to such an ethical review. No such requirements were present for this study.

### Stimuli and Procedure

**FIGURE 1:**
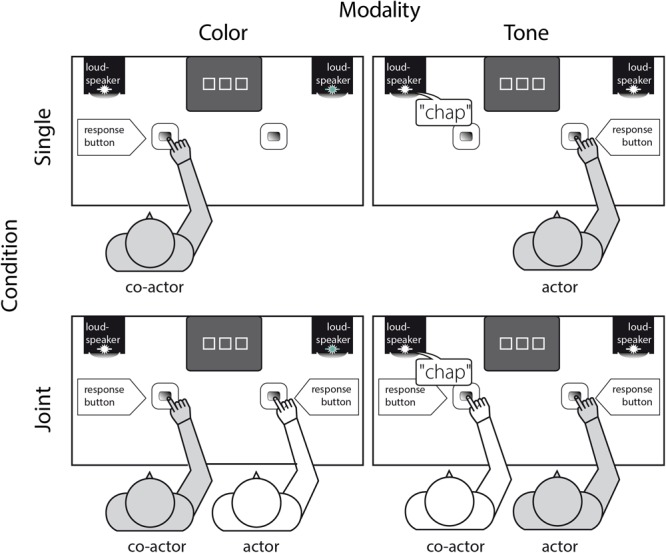
Experimental setup. Gray-shaded person indicates the person responding to his/her assigned stimulus, i.e., either to the visual modality and thus the color “green” (co-actor, left column) or the auditory modality and thus the tone “chap” (actor, right column) - in a stimulus-response incompatible go-trial of the single (upper panel) or the joint condition (lower panel, order of the conditions were counterbalanced across participants). Hence, at a single trial in both the single and joint go-nogo Simon task condition there was only one stimulus presented (i.e., “chap” or a green light) that forced the respective participant to respond (i.e., go-trial) or to withhold from responding in case of a stimulus delivered in the other modality (i.e., nogo-trial).

Only *one auditory* and only *one visual* signal was chosen as go and nogo stimuli in the present bi-modal go-nogo version of the Simon task. The auditory signal consisted of the spoken Dutch color word – “pars” (purple) – played in reverse so that no word was recognizable to our German participants (i.e., “chap”) and presented at approximately 60 dB to either the left or right loudspeaker separated by a distance of one meter (i.e., 50 cm to the left or 50 cm to the right of the midline of the screen). The visual stimulus, a green light, was delivered via the left or the right light emitting diode (LED, *r* = 1 cm) attached on the top of the left and right loudspeaker (exceeding a visual angle of 79.6° × 18.9°; see Figure 1). However, to maintain participants’ fixation at the center of the computer screen, an array of three squares, framed in white on a gray background (10.7° × 2.2°), was presented throughout each trial (i.e., from beginning until response execution), with the middle square serving as the fixation point (2.2° × 2.2°).

Upon arrival at the laboratory, pairs of participants were informed that they would perform the same task in two different conditions, i.e., they would perform the task alone in one condition (i.e., single condition, Figure 1, upper panel) and the same task together with the other person in the other condition (i.e., joint condition, Figure 1, lower panel; see Tsai et al., 2008; Atmaca et al., 2011; Pfister et al., 2014, for the same practice of introducing different experimental condition to the participants).

In the joint condition (Figure 1, lower panel); both participants were seated next to each other. They operated a response button with their right index-finger (25 cm in front and 25 cm from the midline of a 17″ computer monitor) and were asked to place their left hand underneath the table on their left thigh. Prior to the experiment, participants were familiarized with the task, including the presentation of the two stimuli and their assignment as go and nogo stimuli (e.g., “Person on the RIGHT press the response key if you see the green light and person on the LEFT respond by pressing the key if you hear ‘chap”’). The individual target stimulus (auditory, visual), the response side (left, right) and the order of conditions (Single, Joint) were counterbalanced across participants (i.e., half of the participants started with the joint followed by the single condition, while the other half performed both conditions in reversed order).

In the single condition (Figure 1, upper panel), everything was held constant (i.e., assigned stimulus and response side) except that the left or right chair remained empty.

The whole experiment consisted of two consecutive sessions, one single and one joint session, with the order of sessions counterbalanced across participants. Each session comprised three blocks, one training of 2 trials (equals 8) and two experimental blocks of 64 trials for each stimulus (auditory vs. visual) and S-R mapping (compatible vs. incompatible; equals 256 trials). To improve participant vigilance throughout the whole experiment, short breaks between blocks and a 5 min break between conditions outside the laboratory were provided.

Each trial (irrespective of the condition) began with the simultaneous presentation of the square array and a fixation-sound for 300 ms. After 700 ms, the critical stimulus – either the auditory or the visual signal – was presented for 300 ms to the left or the right loudspeaker/LED. Participants were encouraged to respond as quickly and as accurately as possible. After a response was given or 1500 ms had passed, a 1000 ms inter-stimulus-interval (i.e., a blank screen) followed. Note that in the Single go-nogo condition, 1500 ms had to pass in case of a nogo trial before the inter-stimulus-interval started.

## Results

### Reaction Times

For statistical analysis, we excluded all trials in which the responses were incorrect (0.7%), or had a reaction time (RT) less than 150 ms or greater than 1000 ms (1.2%; Röder et al., 2007; Dolk et al., 2011, 2013; Liepelt et al., 2011). Responses were coded as compatible (stimulus ipsilateral to the correct response side) and incompatible (stimulus contralateral to the correct response side). To investigate the SRCs, correct RTs were submitted to an analysis of variance (ANOVA) with Compatibility (compatible, incompatible), and Condition (single, joint) as within-subjects factors and Modality (auditory, visual) as a between-subjects factor.

This 2 × 2 × 2 ANOVA revealed a significant main effect of Compatibility, *F*(1,38) = 95.42, *p* < 0.001, η_p_^2^ = 0.72, showing that responses were faster with stimulus-response compatibility (mean RT = 269 ms, *SD* = 43 ms) than with stimulus-response incompatibility (mean RT = 286 ms, *SD* = 45 ms)^1^. The main effect of Condition was also significant, *F*(1,38) = 8.56, *p* < 0.01, η_p_^2^ = 0.18, showing that responses in the single condition were overall faster (mean RT = 269 ms, *SD* = 46 ms) than in the joint condition (mean RT = 286 ms, *SD* = 41 ms). The main effect of Modality was not significant (*F* < 1).

**FIGURE 2:**
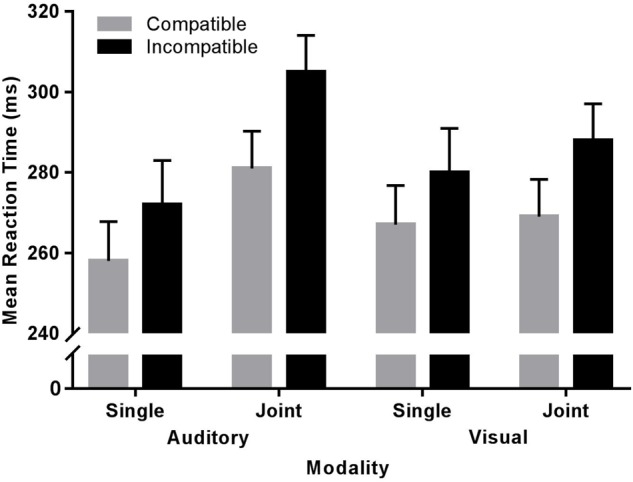
Mean reaction time (ms) as a function of Compatibility (compatible, incompatible), Condition (single, joint) and Modality (auditory, visual). Errors bars represent the standard error (SE).

More importantly, the SRC varied between conditions, as indicated by a significant interaction of Compatibility × Condition, *F*(1,38) = 9.15, *p* < 0.01, η_p_^2^ = 0.19. The step-down analysis by the factor Condition revealed significant SRCs in both conditions, with a 21 ms compatibility effect observed in the joint condition, *F*(1,38) = 90.72, *p* < 0.001, η_p_^2^ = 0.70, and a 14 ms compatibility effect in the single condition, *F*(1,38) = 41.16, *p* < 0.001, η_p_^2^ = 0.51 (Figure 2 and Table 1). Note, this modulation of the SRC by condition as well as the SRC as such was independent of the specific stimulus modality to which participants responded (all *F*s < 1)^2,3^ .

**Table 1:** Mean and standard deviation of reaction time (ms), error rate (%), for compatible and incompatible trials as well as spatial compatibility effect (SRC; compatible minus incompatible trials) as a function of condition (joint, single), and modality (auditory, visual).

	Compatible		Incompatible		SRC	
	*M*	*SD*	*M*	*SD*	*M*	*SD*
	**Reaction time**					
Single*_Auditory_*	258	56	272	61	14^∗^	5
Single*_V isual_*	267	27	280	34	13^∗^	7
Joint*_Auditory_*	281	52	305	47	24^∗^	5
Joint*_V isual_*	269	28	288	33	19^∗^	5

	**Error rates**					
Single*_Auditory_*	0.32	0.82	0.24	0.76	–0.08^†^	–0.06
Single*_V isual_*	0.00	0.00	0.00	0.00	0.00^†^	0.00
Joint*_Auditory_*	0.32	0.66	0.24	0.59	–0.08^†^	–0.07
Joint*_V isual_*	0.95	1.56	0.79	1.39	–0.16^†^	–0.17

*^∗^*p* < 0.001, ^†^not significant.*

However, responses to the auditory modality in the single condition were faster (mean RT = 265 ms, *SD* = 59 ms) compared to the joint modality [mean RT = 293 ms, *SD* = 50 ms; *F*(1,19) = 7.78, *p* < 0.05, η_p_^2^ = 0.29], while this was not the case for responses to the visual modality [mean RT_Single_ = 274 ms, *SD*_Sinlge_ = 31 ms; mean RT_Joint_ = 279 ms, *SD*_Joint_ = 31 ms; *F*(1,19) < 1] as indicated by a significant interaction of Condition × Modality, *F*(1,38) = 4.20, *p* < 0.05, η_p_^2^ = 0.10.

Indicated by one reviewer, the data of both co-actors might not be fully independent. To cope for this, we split the data using the factor modality and ran two separate ANOVAs. However, results did not change (for details, see Table 2).

**Table 2:** Results of separate ANOVAs for the auditory and visual participants.

	Audio	Visual
Compatibility	*F*(1,19) = 83.57, *p* < 0.001, η_p_^2^ = 0.82	*F*(1,19) = 36.37, *p* < 0.001, η_p_^2^ = 0.66
	Compatible: 260 (34) ms	Compatible: 268 (34) ms
	Incompatible: 280 (40) ms	Incompatible: 284 (40) ms
	Simon: 20 ms	Simon: 16 ms
Condition	*F*(1,19) = 7.62, *p* < 0.05, η_p_^2^ = 0.30	*F*(1,19) = 0.93, *p* > 0.05, η_p_^2^ = 0.05
	Single: 265 (59) ms	Single: 274 (30) ms
	Joint: 293 (50) ms	Joint: 279 (31) ms
Compatibility x Condition	*F*(1,19) = 9.03, *p* < 0.01, η_p_^2^ = 0.33	*F*(1,19) = 6.61, *p* < 0.05, η_p_^2^ = 0.26
	*Single: F*(1,19) = 21.84, *p* < 0.01, η_p_^2^ = 0.54,	*Single: F*(1,19) = 18.43, *p* < 0.001, η_p_^2^ = 0.49,
	Compatible: 258 (56) ms	Compatible: 267 (27) ms
	Incompatible: 272 (61) ms	Incompatible: 280 (34) ms
	Simon: 14 ms	Simon: 13 ms
	*Joint: F*(1,19) = 43.75, *p* < 0.001, η_p_^2^ = 0.70,	*Joint: F*(1,19) = 49.86, *p* < 0.001, η_p_^2^ = 0.72,
	Compatible: 281 (52) ms	Compatible: 269 (28) ms
	Incompatible: 305 (47) ms	Incompatible: 288 (33) ms
	Simon: 24 ms	Simon: 19 ms

### Error Rates

The 2 × 2 × 2 ANOVA revealed a significant main effect of Condition, *F*(1,38) = 7.61, *p* < 0.01, η_p_^2^ = 0.17, indicating that participants made more errors when performing the task together with another person (0.6%) compared to when working alone (0.2%). This effect was varied as a function of Modality, *F*(1,38) = 7.44, *p* < 0.05, η_p_^2^ = 0.16, showing that participants made more errors in response to auditory compared to visual stimuli in the single condition (0.3% vs. 0.0%) but the reverse was true in the joint condition (0.3% vs. 0.9%). No other effects or interactions reached significance (all *F*s < 1).

## Discussion

The aim of the present study was to investigate the effect of alternative stimulus events in the absence (single task) or presence (joint task) of alternative action events on task performance. When participants responded to stimuli in a single sensory modality and withheld responses to stimuli in another modality, we found reliable SRCs in both the single and the joint go-nogo Simon task condition (single < joint), for both visual and auditory sensory modalities. This finding contradicts the assumption that reliable go-nogo SRCs in the single go-nogo condition are restricted to cases in which there is correspondence between the modality of stimulus material and attention-grabbing alternative events. Rather, the present findings suggest that the spatial coupling of alternative events, here accomplished by presenting auditory and visual stimuli in the same locations, facilitates their integration, and thus creates the need to discriminate between them in order to respond appropriately in a given context. The finding of such integration is in line with multisensory research showing that the processing of spatial stimuli coming from different sensory modalities seems to rely on a shared pool of attentional resources (Wahn and König, 2017). When the task of responding to events coming from visual and tactile modalities is distributed across two persons, the crossmodal congruency effect was found to be socially modulated (Heed et al., 2010). However, in contrast to our finding of an increased SRC in the joint as compared to a single go-nogo Simon task condition, Heed et al. (2010) observed a significantly reduced crossmodal congruency effect under joint as compared to single conditions. This reduction was mainly due to faster performance on incongruent trials. One might attribute these different findings to different modality combinations used across these studies – visual-auditory in our study vs. visual-tactile in the study of Heed et al. (2010). However, a more recent study by Wahn et al. (2017) showed a similar reduction of the joint crossmodal congruency effect with an audio-visual crossmodal congruency task. Thus, an effect of different modality pairings is unlikely to explain this discrepancy. Instead, the opposite effects between the Heed study and our study are more likely to be attributed to different task demands (Liepelt and Fischer, 2016) and whether the joint task allows a division of labor or not. When a division of labor across persons is possible, the burden or distraction of alternative event representations is reduced (cf. Sellaro et al., 2013, 2018). In the present study, however, the discrimination of alternative events cannot be handed over to the partner and thus cannot be separated. On each trial a discrimination has to be performed in order to either go or withhold the response. Thus, in the present study the need to discriminate between these events is an additional demand, explaining the increase in reaction time in the joint as compared to the single-task condition (cf. Yamaguchi et al., 2018a). Furthermore, our findings relate to a study showing that peripersonal space boundaries shrink when subjects face another individual (Teneggi et al., 2013). During joint action it has been shown that attention to items appearing in the peripersonal space and intentional weighting interact, so that the effect of enhanced spatial processing for those items is counteracted by a stronger weighting of discriminative action features (Liepelt, 2014), thus increasing the Simon effect.

In previous work using tasks that require performance of selective (i.e., go-nogo) responses to different features within the same sensory modality (e.g., auditory, tactile and/or auditory sensation), SRCs are typically observable in the presence (i.e., “joint” condition) of (social or non-social) reference-providing events in the response dimension, but not when those attention-grabbing events are absent (i.e., single condition; Sebanz et al., 2003; Dolk et al., 2011, 2013; for a review, see Dolk et al., 2014). The present findings extend this body of work by indicating that stimuli presented in different sensory modalities influence information processing and response selection not only when jointly performing such complementary multimodal go-nogo Simon task, but even in the absence of any perceivable reference-providing event in the response dimension, viz. the single go-nogo condition (Stenzel and Liepelt, 2016). Additionally, this finding provides further evidence against the notion of action and/or task co-representation (Atmaca et al., 2011; Sebanz et al., 2003)^4^, thereby calling for an alternative explanation (for a review, see Prinz, 2015).

Sudden onsets of stimulus events in two different modalities that call for distinct, corresponding (spatially defined) action alternatives – to act/go or not to act/nogo – may inevitably direct attention to features that enable perceptual discrimination in the stimulus domain. Given that this (stimulus) event discrimination is typically followed by perceivable consequences of spatially related action alternatives (cf. Baess and Prinz, 2015)^5^ (Milanese et al., 2010, 2011; Iani et al., 2014), discriminable features can increase the weight of codes on which their cognitive representation is determined (Hommel et al., 2001; Memelink and Hommel, 2013). As stimulus events in the Simon tasks are typically coupled with particular action events, the tight spatial and temporal co-occurrence of perceptual (i.e., stimulus and action) events leads to the transient, episodic integration of the respective features into *event-files, object-files*, or *object tokens* (Kahneman et al., 1992; Schneider, 1995; Hommel et al., 2001, respectively).

Consequentially, strengthening one member of these cognitive bindings through intentional weighing (or the distribution of attentional weights thereupon; Bundesen, 1990; Schneider, 1995) may influence the activation of other members involved in such bindings, such as the spatial features that discriminate their subsequent responses from other events in the Simon task (Hommel et al., 2001; Memelink and Hommel, 2013). The activation strength of specific features depends upon whether and how strongly the dimension of features is defined by task-relevance and task setting. In the present study, the sensory stimulus modality (auditory/visual), the size of scope of the attentional focus, and spatially pre-defined action alternatives (left/right) seem to be important dimensions receiving the most weight in the event-file.

In other words, making the representation of alternative stimulus and action events more task-relevant – by emphasizing the coding of discriminable features via stimulus processing – increases the competition between these representations as well as those events associated and spatially/temporally coupled with them. Based on the experimental setting of the present study, this means that these representations involve sensory features according to the specific stimulus modality and spatial features of the to-be-executed action alternatives, which induces at least two different competitions between feature codes (Duncan, 1996; Dutzi and Hommel, 2009). Given that response selection can only proceed when stimulus events have successfully been dissociated, reaction time should increase with every extra feature dimension that is considered in the process of event-coding in go-nogo settings (single > joint, see Figure 2). Accordingly, in contrast to previous findings of (social) SRCs, the present results provide no indication for social facilitation when sharing a multimodal Simon task with another person. Instead, and in line with the presented framework, additional action events that need to be discriminated in the course of response coding further signified the task-relevance of nogo stimuli, thereby providing an explanation for the further increase of SRCs from single go-nogo to joint go-nogo conditions, a process that has been termed nogo-/inhibitory tagging (Liepelt et al., 2011).

From a mechanistic perspective, stimulus events in the Simon task are widely accepted to exert their impact on response competition mainly via task-irrelevant (i.e., spatial) features. This results either in the activation of the same (compatible trials) or the opposite (incompatible trials) response leading to facilitation or interference, respectively. This impact of competing event representations should be even stronger if the significance of task-relevant stimulus features (i.e., via the multi-modality) highlights the corresponding (spatially defined) action alternatives. This seems to hold irrespective of whether the action is to-be-executed or not (cf. Kühn and Brass, 2010a,b) and even more relevant when alternative stimulus events share locations of possible occurrences (Stenzel and Liepelt, 2016; Puffe et al., 2017). In prior work, stimulus events and attention-grabbing alternative (action) events were spatially distinct and influential only in cases of a (modality) correspondence between stimulus and response event (e.g., Dolk et al., 2013; Lien et al., 2016; Puffe et al., 2017). In the present experiment, the spatial overlap of both the relevant feature dimension of the stimuli and the alternative stimulus event in single and joint go-nogo Simon task conditions seems to challenge go-nogo decisions reliably. In the joint condition, where associated action events are to be distinguished on top of the perceptual discrimination via the stimulus modality, the task relevance of those go-nogo decisions can be considered to be further strengthened, thereby providing an explanation for the significantly increased SRC in the joint condition.

In sum, although a spatial and temporal correspondence of stimulus material and attention-grabbing event might be an efficient condition for SRCs to emerge, the driving force underlying the emergence of SRCs rather appears to be (the width of) the attentional focus that either prevents or facilitates alternative events to be integrated and therefore requiring discrimination from task-relevant events. This assumption is in line with previous findings showing reliable SRCs in the single go-nogo condition or even enlarged SRCs in the presence of (non-/social) action events when: (i) attentional capacities are available to integrate alternative events (e.g., Dolk et al., 2013; Lien et al., 2016; Puffe et al., 2017), (ii) all perceivable events are in the focus of attention (e.g., Stenzel and Liepelt, 2016), (iii) attention is directed toward the space of alternatives by acting upon those directly (e.g., Porcu et al., 2016), or (iv) current cognitive states attenuating or enlarging the attentional focus (Colzato et al., 2012a,b). Thus, as soon as the attentional focus is broad enough to enable the integration and cognitive representation of alternative events, the difficulty of discriminating between events that are concurrently active is increased by any additional stimulus or response event challenging this process. The results of this are reliable SRCs in single and “joint” go-nogo Simon tasks.

## Author Contributions

All authors listed have made a substantial, direct and intellectual contribution to the work, and approved it for publication.

## Conflict of Interest Statement

The authors declare that the research was conducted in the absence of any commercial or financial relationships that could be construed as a potential conflict of interest.

## Footnotes

1 To provide the reader with a baseline effect, we run the same experiment with 10 new subjects (6 female; *M*_age_ = 24.1, *SD*_age_ = 3.3, *R*_age_ = 20–31 years) in the standard two-choice version. Results revealed a significant SRC effect (33 ms), *F*(1,9) = 24.72, *p* < 0.01, η_p_^2^ = 0.73, showing that responses were faster with stimulus-response compatibility (mean RT = 398 ms, *SD* = 73 ms) than with stimulus-response incompatibility (mean RT = 431 ms, *SD* = 64 ms).

2 To rule out any effect of the order of conditions (single and Joint), we included Order as a between-subjects factor into the 2 × 2 × 2 ANOVA (Compatibility, Condition, Modality). The respective 2 × 2 × 2 × 2 analysis revealed no significant four-way interaction, *F*(1,36) = 2.16, *p* = 0.150, η_p_^2^ = 0.06, suggesting that the order had no influence on the observed overall pattern of results. However, given that Modality overall had no influence on the emergence of SRCs in single and joint conditions, one might still wonder as to whether the order of conditions might influence the SRCs independent of Modality. A respective 2 × 2 × 2 ANOVA revealed no significant interaction between Compatibility, Condition, and Order, *F*(1,38) = 3.66, *p* = 0.063, η_p_^2^ = 0.09. For the sake of completeness, however, we still performed an additional step-down analysis by order. While the Compatibility × Condition interaction for those who started with the single go-nogo condition did not reach significance, *F*(1,19) = 0.61, *p* = 0.443, η_p_^2^ = 0.031, this interaction was significant for those who started with the joint go-nogo condition, *F*(1,19) = 16.65, *p* = 0.001, η_p_^2^ = 0.47. Note, however, that although the go-nogo SRC effect in the single condition was significantly smaller [*t*(19) = 4.64, *p* < 0.001] as compared to the joint condition [*t*(19) = 8.74, *p* < 0.001] the SRC effect was reliable across both single [*t*(19) = 4.47, *p* < 0.001] and the joint [*t*(19) = 5.38, *p* < 0.001] tasks in both groups. Thus, even though there is some variation depending on the order of conditions, the overall pattern of a reliable SRC in the single and joint go-nogo condition is consistent.

3 As requested by one reviewer we now provide an additional bin analyses in order to shed more light on the temporal dynamics of the multimodal SRC. To that end, we computed, separately for each condition and participant, the RT distributions, which we divided into four bins (quartiles). These data were analyzed by means of an ANOVA with condition, compatibility, bin, and modality as factors. A respective 2 × 2 × 4 × 2 ANOVA with Bin (1,2,3,4) as additional within-subjects factor revealed no significant four-way interaction (*F* < 1). These results clearly provide no evidence in favor of a modality-driven difference in the time course of the go-nogo Simon effect different to what is often observed in the two-choice Simon task (for more discussion on the issue, see Wascher et al., 2001; Leuthold and Schröter, 2006; Xiong and Proctor, 2016; D’Ascenzo et al., 2018). As to whether these results indicate a further example for the difference, rather than the similarity of two-choice and go-nogo Simon task is an interesting topic that warrants further investigation.

4 A similar pattern of results was already shown by Sebanz et al. (2005) who forced one participant to respond to the pointing direction of the stimulus hand whereas the other person had to respond to a colored ring attached to the stimulus hand (Sebanz et al., 2005). Counterbalancing single and joint go-nogo conditions of this task across participants revealed a SRC in the joint, but most interestingly also a reliable effect in the single go-nogo condition. Even though the authors described the latter finding more as an accident, i.e., as an compatibility effect in its own right (see Hommel, 1996) or due to an carryover effects from joint to single conditions (Sebanz et al., 2005), it highlights the so far widely underestimated impact of stimulus feature (i.e., attentional breadth) on information processing and response selection that clearly warrant further investigations.

5 Based on recent findings of Kühn and Brass (2010a,b), who showed that with-holding an action (e.g., a nogo due to instruction) is explicitly and more importantly represented as action-specific, we expect an instruction “not to act” to be cognitively represented as a simple alternative to one’s own action event representation. Thus, withholding a response does not necessarily need the perception of such an alternative (as long as it refers to a comparable action event) to activate its sensory consequences.
